# In Memoriam: Daniel L. Kastner, MD (1951–2026)

**DOI:** 10.70962/jhi.20260153

**Published:** 2026-09-11

**Authors:** Sudhir Gupta, Raphaela Goldbach-Mansky, Ivona Aksentijevich

**Affiliations:** 1 University of California, Irvine, Irvine, CA, USA; 2 National Institute of Allergy and Infectious Diseases, National Institutes of Health, Bethesda, MD, USA; 3 National Human Genome Research Institute, National Institutes of Health, Bethesda, MD, USA

## Abstract

Dr. Daniel L. Kastner, former Scientific Director of the National Human Genome Research Institute (NHGRI), was a visionary physician-scientist whose discoveries transformed the understanding of innate immunity and established the modern field of autoinflammatory diseases. Early in his career, he led the International FMF Consortium in identifying mutations in the *MEFV* gene as the cause of familial Mediterranean fever, providing the first molecular basis for a hereditary periodic fever syndrome and fundamentally redefining the pathogenesis of inflammatory disease. He subsequently identified and characterized additional hereditary autoinflammatory disorders, developed the conceptual framework for autoinflammation, and demonstrated how the study of rare genetic diseases could reveal universal mechanisms governing human inflammation and immunity. As Chief of the Inflammatory Disease Section at the National Institute of Musculoskeletal and Skin Diseases and later at the NHGRI, he built an internationally recognized program that integrated clinical investigation, human genetics, and immunology to advance both scientific discovery and patient care. Dr. Kastner mentored generations of physician and scientists and inspired colleagues through his intellectual rigor, humility, wit, and generosity. He will be remembered as the founder of the field of autoinflammatory diseases, an extraordinary translational investigator, a dedicated mentor, and a compassionate physician whose discoveries transformed the lives of patients worldwide.

## Teaser

Dr. Daniel L. Kastner, former Scientific Director of NHGRI, was a visionary physician-scientist whose discoveries transformed the understanding of innate immunity and established the modern field of autoinflammatory diseases. He led the International FMF Consortium in identifying mutations in the *MEFV *gene as the cause of familial Mediterranean fever.

The field of human immunology has lost one of its most influential pioneers with the passing of Daniel L. Kastner, MD. His visionary discoveries transformed our understanding of the innate immune system and established the modern field of autoinflammatory diseases.

**Figure 1:**
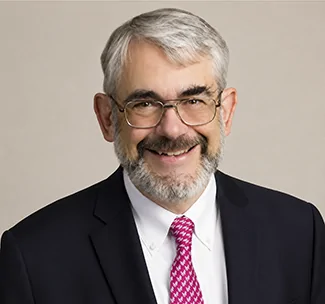
Daniel L. Kastner. Courtesy of Sudhir Gupta.

Through elegant clinical investigation integrated with human genetics, Dr. Kastner demonstrated that dysregulation of innate immunity could give rise to a distinct class of inflammatory disorders, fundamentally reshaping concepts of immune-mediated disease. His landmark discovery that mutations in the *MEFV* gene cause familial Mediterranean fever (1), followed by the identification and characterization of numerous hereditary autoinflammatory syndromes, laid the foundation for an entirely new discipline with in immunology. The concept of autoinflammatory diseases, introduced in 1999, was subsequently expanded to encomplass a growing spectrum of disorders and thier underlying molecular mechanisms (2). His laboratory continued to make seminal contributions to the field, including elucidating the mechanisms of inflammasome regulation and evolutionary significance of *MEFV* mutations (3), identifying deficiency of adenosine deaminase 2 (DADA2) and vacuoles, E1 enzyme, X-linked, autoinflammatory, somatic syndrome as novel autoinflammatory disorders (4, 5), and advancing mechanism-based therapies for DADA2 and other diseases (6). Together, these discoveries revolutionized the diagnosis and treatment of patients with recurrent inflammatory disorders while illuminating fundamental mechanisms governing inflammation.

As Chief of the Inflammatory Disease Section and Scientific Director of the National Human Genome Research Institute from 2010 to 2021, Dr. Kastner cultivated an extraordinary environment, where clinical observation, genetics, and immunology converged to advance both science and patient care. His work exemplified the power of translational medicine, demonstrating how careful study of rare diseases could reveal universal principles of human biology.

Dr. Kastner’s groundbreaking contributions were recognized with many of the highest honors in biomedical science, including election to the National Academy of Sciences, the 2024 Crafoord Prize for pioneering discoveries that established the field of autoinflammatory diseases, the Ross Prize in Molecular Medicine, the American College of Rheumatology Presidential Gold Medal, the Thomas A. Waldmann Award for Excellence in Human Immunology, and the Samuel J. Heyman Service to America Medal as Federal Employee of the Year. These honors reflected not only the profound scientific impact of his work but also its transformative influence on the care of patients around the world.

Beyond his scientific achievements, Dr. Kastner was an exceptional mentor, physician, and colleague. His intellectual curiosity, scientific rigor, wit, and generosity inspired generations of investigators who now continue to expand the field he helped create. His stature in the field never made others feel small**;** rather, it encouraged others to aim higher. This combination of excellence and humility is a rare gift to possess. Dan’s influence extends far beyond his remarkable body of publications through the countless trainees, collaborators, and patients whose careers and lives he shaped.

Dr. Kastner was also a founding member of the Scientific Advisory Board of the *Journal of Human Immunity*, where his insight, wisdom, and unwavering commitment to scientific excellence helped shape the journal from its inception.

The impact of Dr. Kastner’s work is immeasurable. His discoveries transformed the lives of patients worldwide, established new paradigms in immunology, and continue to guide the development of precision therapies for inflammatory diseases. Few physician-scientists have so profoundly improved both scientific understanding and clinical practice.

We have lost a friend. The *Journal of Human Immunity* joins the international immunology community in mourning the loss of an extraordinary physician-scientist whose legacy will endure through the science he created, the patients he served, and the generations of investigators he inspired. We extend our deepest condolences to his wife of 41 years, Margaret Beckwith; his sons, Benjamin and Nathan; his daughter-in-law, Jamie; and his many friends, colleagues, collaborators, and former trainees.

Daniel L. Kastner’s contributions to human immunology will remain a lasting source of knowledge and inspiration for decades to come.
